# The development of a mini-array for estimating the disease state of gastric adenocarcinoma by array CGH

**DOI:** 10.1186/1471-2407-8-393

**Published:** 2008-12-30

**Authors:** Tomoko Furuya, Tetsuji Uchiyama, Atsushi Adachi, Takae Okada, Motonao Nakao, Atsunori Oga, Song-Ju Yang, Shigeto Kawauchi, Kohsuke Sasaki

**Affiliations:** 1 Department of Pathology, Yamaguchi University School of Medicine, Ube 755-8505, Japan; 2 Department of Surgery, Iwakuni Medical Center, Iwakuni 740-0021, Japan; 3 Macrogen Inc, Seoul 153-081, Republic of Korea

## Abstract

**Background:**

The treatment strategy usually depends on the disease state in the individual patient. However, it is difficult to estimate the disease state before treatment in many patients. The purpose of this study was to develop a BAC (bacterial artificial chromosome) mini-array allowing for the estimation of node metastasis, liver metastasis, peritoneal dissemination and the depth of tumor invasion in gastric cancers.

**Methods:**

Initially, the DNA copy number aberrations (DCNAs) were analyzed by array-based comparative genomic hybridization (aCGH) in 83 gastric adenocarcinomas as a training-sample set. Next, two independent analytical methods were applied to the aCGH data to identify the BAC clones with DNA copy number aberrations that were linked with the disease states. One of the methods, a decision-tree model classifier, identified 6, 4, 4, 4, and 7 clones for estimating lymph node metastasis, liver metastasis, peritoneal dissemination, depth of tumor invasion, and histological type, respectively. In the other method, a clone-by-clone comparison of the frequency of the DNA copy number aberrations selected 26 clones to estimate the disease states.

**Results:**

By spotting these 50 clones together with 26 frequently or rarely involved clones and 62 reference clones, a mini-array was made to estimate the above parameters, and the diagnostic performance of the mini-array was evaluated for an independent set of 30 gastric cancers (blinded – sample set). In comparison to the clinicopathological features, the overall accuracy was 66.7% for node metastasis, 86.7% for liver metastasis, 86.7% for peritoneal dissemination, and 96.7% for depth of tumor invasion. The intratumoral heterogeneity barely affected the diagnostic performance of the mini-array.

**Conclusion:**

These results suggest that the mini-array makes it possible to determine an optimal treatment for each of the patients with gastric adenocarcinoma.

## Background

Gastric adenocarcinoma is one of the most frequent cancers worldwide, and it is the second leading cause of cancer death [1]. Although new diagnostic and therapeutic procedures are being developed, many patients still nevertheless die of the disease. The improvement of prognosis by the most optimal treatment is the first priority for all cancer patients. It is true that detection is critical to a rapid cure, though under the present circumstances gastric cancers are detected at various stages. The treatment strategy usually depends on the disease state in each individual patient. Surgery with a curative intent is applied to advanced cancers, while an endoscopic mucosal resection (EMR) or endoscopic submucosal dissection (ESD) is applied to early cancer without lymph node metastasis [2]. Accordingly, it is necessary to elucidate the disease state of an individual patient before any treatment or therapeutic procedure is started. In particular, an accurate evaluation of the nodal status and disease stage is critical in order to determine the appropriate treatment. However, the assessment of the disease state before treatment is not easy by conventional tests such as endoscopic inspection and microscopic examination of biopsy specimens. It is generally accepted that the biological characteristics of cancer are primarily dependent on the underlying genetic alterations of cancer cells. Therefore, a comprehensive analysis of the genomic changes in an individual type of cancer is necessary for identifying the genomic changes linked with the clinicopathological features. Microarray technology accomplishes this purpose. Array-based comparative genomic hybridization (aCGH), a specific microarray method, allows the locus-by-locus measurement of DNA copy number aberrations (DCNAs) in cancer cells with a high resolution [3]. The aCGH has been applied to surgically removed gastric cancers to identify the chromosomal regions associated with carcinogenesis [4-9]. However, information concerning the relationship between DCNAs and the disease state is currently very limited. The identification of BAC clones with DCNAs that are linked with disease states may allow the exact estimation of disease states even in biopsy specimens at the time of the histological diagnosis, or before treatment. When a mini-array as a gastric cancer classifier that makes possible the quantitative measurement of genomic alterations is developed, it will be possible to estimate the disease state of each tumor for optimal treatment. The mini-array CGH may provide information concerning the disease states in order to determine the optimal treatment in individual cancer patients, thus contributing to personalized gastric cancer care.

In this study, the statistical analyses of aCGH data obtained from 83 sporadic gastric cancers identified 50 BAC clones linked with disease states including node metastasis, liver metastasis, peritoneal dissemination, the depth of tumor invasion and the histological type in gastric cancers. Next, using these BAC clones, a mini-array was constructed to evaluate the disease states of gastric cancer. In addition, the diagnostic performance of the mini-array was evaluated for an independent set of 30 gastric cancers. This is the first report of the development of a BAC mini-array which thus makes it possible to estimate the disease state in gastric cancer.

## Methods

### Tissue specimens and DNA samples

Eighty-three consecutive surgically removed gastric adenocarcinomas were evaluated for the selection of BAC clones linked with the clinicopathological features. They included 10 early cancers and 73 advanced cancers. The patients consisted of 62 males and 21 females with an average age of 70 years ranging from 44 to 89 years old. The family histories were noncontributory for all patients and all tumors were considered to be sporadic. The clinicopathological features are summarized in Table 1. In brief, according to Lauren's histological classification, 41 tumors were classified as intestinal-type gastric cancer, while the others were diffuse-type cancers. Node and liver metastases were detected in 60 and 6 cancers, respectively. In this series, 19 tumors showed a peritoneal dissemination of the cancer cells. The tissue specimens were stored at -80°C until use. A tissue microdissection technique was used to reduce the contamination of the normal tissue components for the array CGH analyses, as previously described [10]. High molecular weight genomic DNA was extracted from the microdissected tumor tissue specimens with a DNA extraction kit (SepaGene, Sankojunyaku Co., Ltd, Tokyo, Japan) according to the manufacturer's instructions [11-13]. Control DNA (Promega, Madison, WI) was used as a reference. The study protocol was conducted under the approval of the Institutional Review Board for Human Use at the Yamaguchi University School of Medicine in 2004, and informed consent for this study was obtained from every patient.

**Table 1:** Clinicopathological summary of 83 gastric adenocarcinomas

**For Screening**		
The number of gastric cancers examined:	83	
Average age of patients (range):	70 years old (44 – 89 years)	
		
Histological type of gastric cancers		
Intestinal-type:	41	
Diffuse-type:	42	
	Intestinal-type	Diffuse-type
Average age (years)	72	68
Sex (F/M)	9/32	12/30
Node metastasis	26	34
Liver metastasis	3	3
Peritoneal dissemination	7	12
Early/advanced cancers*	6/35	4/38
		
**For validation of the mini-array**		
The number of gastric cancers examined:	30	
Average age of patients (range):	69.3 years old (44 – 88 years)	
Sex (F/M)	11/19	
Histological type of gastric cancers		
Intestinal/diffuse	13/17	
Node metastasis	9	
Liver metastasis	3	
Peritoneal dissemination	5	
Early/advanced cancers*	3/27	

*Early gastric cancer is defined as a tumor with invasion limited to mucosa or submucosa according to the Japanese classification of gastric carcinoma (9).

### Array CGH for screening

The array CGH experiments were performed with a MacArray™ Karyo 1400 (Macrogen Inc., Seoul, Korea) according to the manufacturer's protocol , which provided the BAC chip information together with information of the end-sequenced BAC clones and data processing methods, as previously described [14-16]. The arrays consisted of triplicate spotted 1,440 human bacterial artificial chromosome (BAC) clones, including 356 cancer-related genes, which covered the whole human genome at an average interval of 2.3 Mb. Sample and gender matched reference genomic DNAs (500 ng each) were labeled by the random priming method with fluorescence dyes, Cy 3 and Cy 5, respectively. The labeled DNAs were mixed with Cot-1 DNA (50 μg, Gibco BRL, Gaithersburg, MD) and then were hybridized to the array slides for 2 days at 37°C in a moist chamber. The array slides were rinsed in a washing buffer and dried well. The array slides were scanned with a Gene Pix 4000A scanner (Axon Instruments, Union City, CA). The fluorescence images were analyzed using the MAC Viewer™ software program (Macrogen Inc.) optimized for the analysis of the array as previously reported [14-16]. The fluorescence spots were defined with the automatic grid feature and adjusted manually. All CGH ratios were automatically converted to log base 2. The ratios of the fluorescence intensities of all spots were plotted against the distance of the clones along the chromosomes. For each BAC clone, the average ratios that deviated significantly from zero were considered to be abnormal (± log_2 _0.25), and almost all spots were within the area between the cutoff lines (± log_2 _0.25) for male/female DNA samples. In addition, we defined log_2 _ratio >1.0 as amplifications.

### Array CGH data analysis

The aCGH data of the 83 gastric cancers were analyzed by two independent methods referred to as method 1 and method 2 for convenience. In method 1, a WEKA decision-tree model classifier, J48 [17] was applied to the aCGH data to identify the BAC clones and their copy numbers for differentiating between gastric cancers with and without node metastasis, with and without liver metastasis, with and without peritoneal dissemination, between early and advanced cancers, and between intestinal type and diffuse type cancers. In method 2, the clone-by-clone frequency of the DCNAs between the two groups, *e*.*g*., between cancer with and without node metastasis, was compared using the χ^2^-test to identify the BAC clones that were used for the distinction between two groups. The difference was considered significant when its P-value was less than 0.05, and the BAC clones with a P-value of less than 0.01 were used for the fabrication of a mini-array specific for gastric cancer.

### Customization of BAC mini-array specific for gastric cancer

**Figure 1:**
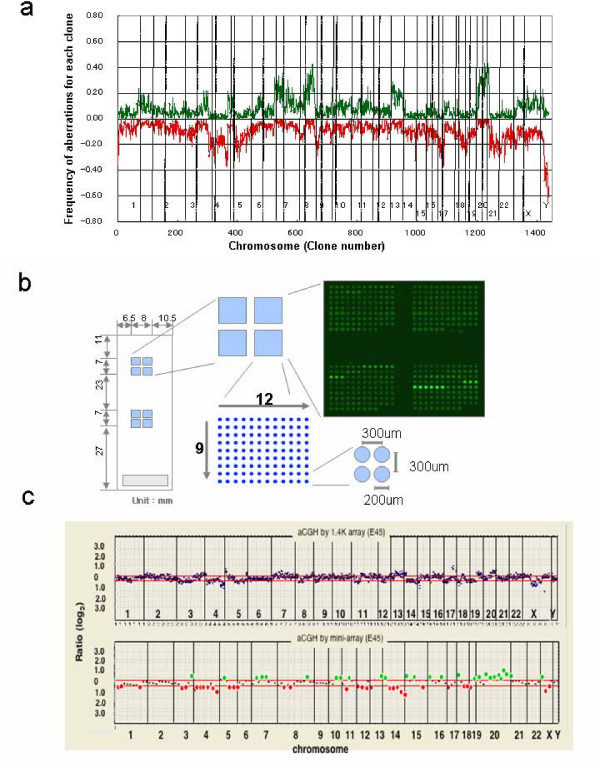
**(a) Frequency of gains and losses in 83 gastric cancers**. A green line denotes a copy number gain and a red line denotes a copy number loss. Gains of 7p, 8q, and 20q, and losses of 4q, 17p, and 21 are frequent in gastric adenocarcinoma. (b-left) Design of a mini-array. The mini-array spotted with 50 BAC clones chosen by two analytical methods of the aCGH data from 83 gastric cancers to estimate the node and liver metastases, peritoneal dissemination, depth of tumor invasion, and histological type, 26 clones with frequent or infrequent DCNAs, and 62 reference clones. A total of 138 BAC clones were spotted in triplicate on two discrete parts of a glass slide, and therefore, each slide was used for two specimens. The diameter of each spot is approximately 200 μm. (b-right) A hybridization image for the mini-array. Note the same color image for three spots each in the mini-array. (c) A comparison of aCGH profiles between the screening chip (upper) and the mini-array (lower) for a case of gastric adenocarcinoma. A profile of aCGH in the mini-array basically replicates that in the screening array. The red lines indicate log_2 _0.25 and -log_2 _0.25, respectively. The green and red spots indicate the BAC clones with a copy number gain and loss, respectively.

The mini-array was made using 50 BAC clones chosen by the two analytical methods of the aCGH data from 83 gastric cancers to estimate node and liver metastases, peritoneal dissemination, depth of tumor invasion, and histological type; the method 1 identified 24 BAC clones of which one was shared between node metastasis and liver metastasis, and the method 2 identified 26 clones. In addition to these BAC clones, the mini-array also contained 26 clones with frequent or infrequent DCNAs and 62 reference clones. A total of 138 BAC clones were spotted in triplicate on two discrete parts of a glass slide, and therefore, each slide was used for two specimens (Fig 1b).

### Validation of performance for mini-array

The performance of the mini-array was evaluated for node metastasis, liver metastasis, peritoneal dissemination, depth of invasion, and histological type of gastric cancers for an independent set of 30 gastric cancers (Table 1). The cutoff value was also applied to data analysis of the mini-array. The copy numbers for 138 BAC clones were compared between the screening arrays and the mini-arrays. When the diagnosis was different between the two methods, priority was given to the diagnosis that indicated a more advanced stage in the evaluation of the mini-array.

### Effects of intratumoral heterogeneity on the performance of mini-array

The effects of the intratumoral genomic heterogeneity on the performance of the mini-array in the randomly selected five gastric tumors were examined. Four tissue specimens were taken from different parts of a tumor. The procedures for tissue microdissection, DNA extraction, aCGH and the data analysis were as noted above.

## Results

The DCNAs were detected by aCGH for the multiple BAC clones in all gastric adenocarcinomas. Although the number of BAC clones with DCNA varied from tumor to tumor, roughly 15% of clones showed DCNAs for each tumor. Overall, frequent DNA copy number gains were detected for chromosomal regions 20q12-q13 (at the frequency of 43%), and 8q24 (43%), and frequent DNA copy number losses were detected for chromosomal regions 4q35.2 (37%), 1p36 (36%), 4q34 (36%), 4q12 (35%), 14q32 (33%), and 22q11 (34%), in addition to the Y chromosome (Fig 1a and see Additional file 1).

**Figure 2:**
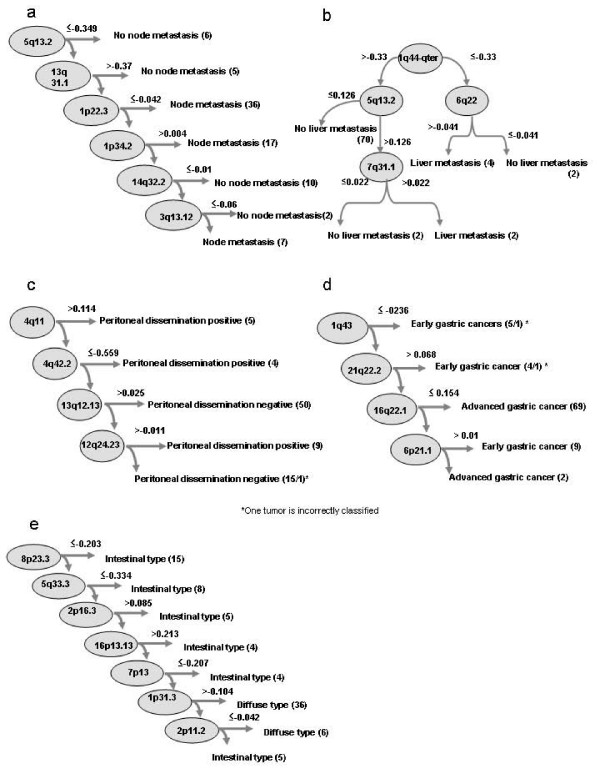
**Identification of BAC clones by a decision-tree algorithm to classify the gastric cancers into two groups with different characters**. (a) Differentiation between gastric cancers with and without node metastasis. These two groups can be clearly differentiated by examining the degree of the copy number changes of six BAC clones mapped to 5q13.2, 13q31.1, 1p22.3, 1p34.2, 14q32.2, and 3q13.12 in descending order. A tumor of which the copy number at 5q13.2 was ≤ -0.346 (log_2_) shows no node metastasis. When the DNA copy number of the tumor was not the case at the first clone, the second criterion (>-0.37 at 13q31.1) was checked. Five tumors were separated from the remaining 77 tumors at this point. When the DNA copy number of the clone at 13q31.1 was ≦ 0.37 in the tumor, the third clone located on 1p22.3 was examined. If the DNA copy number of the tumor meets the criterion of the third clone, ≦ -0.042, nodal metastasis was positive in this tumor. Thirty-six cancers were classified into this category at this point. In this way, the DNA copy number of the tumor was in turn examined from the first to the sixth clone. Each step successively sorts a cluster of either group. Eventually, all gastric cancers were classified into either of two groups, cancers with and without node metastasis. The figures in parentheses indicate the number of tumors fitting the requirements. The correctly classified instances were 83 (100%), and the incorrectly classified instances were 0 (0%). In this classifier, the number of leaves is seven, and the size of the tree was 13. In the same way as the case of node metastasis, BAC clones and their copy numbers were determined for liver metastasis (b), peritoneal dissemination (c), depth of tumor invasion (early or advanced cancer)(d), and histologic type (e).

The comparison of the aCGH data with the clinicopathological features of the tumors using the decision-tree classifier (method 1) allowed the identification of the BAC clones for the differentiation between gastric cancers with and without specific clinicopathological characters. The gastric cancers with node metastasis were distinguished from those without it with an accuracy of 100% by examining the extent of the DCNAs for the six BAC clones mapped to 5q13.2, 13q31.1, 1p22.3, 1p34.2, 14q32.2, and 3q13.12 (Fig 2a). The procedures of the analysis were as follows: The DNA copy number of a tumor at 5q13.2 was applied to the criterion of the first clone. If the DNA copy number of the clone was ≦ -0.349, the tumor was classified as a tumor without node metastasis. Six of 83 tumors were classified into this group. When the DNA copy number of the tumor was not the case at the first clone, the second criterion (>-0.37) was checked. Five tumors were separated from the remaining 77 tumors. When the DNA copy number of the clone at 13q31.1 was ≦ 0.37 in the tumor, then the third clone located on 1p22.3 was examined. If the DNA copy number of the tumor met the criterion of the third clone, ≦ -0.042, nodal metastasis was considered positive in this tumor. Thirty-six cancers were classified into this category. In this way, the DNA copy number of the tumor was in turn examined from the first to the sixth clone. Eventually, all gastric cancers were classified into either of the two groups, cancers with and without node metastasis.

Gastric adenocarcinomas with liver metastasis were separated from those without it at a 100% accuracy rate by checking the DNA copy number for the four BAC clones mapped to 1q44-qter, 5q13.2, 6q23.2, and 7q31.1, as shown in Fig 2b. The high level loss of 5q13.2 was detected in tumors with neither node nor liver metastasis. The decision-tree classifier also permitted the classification of gastric cancer into either a tumor with or without peritoneal dissemination at the correct classification rate of 98.8% by the copy number alterations of the four BAC clones mapped to 4q13.3, 4q32.2, 13q12.13, and 12q24.23 (Fig 2c). The decision-tree classifier made it possible to divide the gastric cancers into two groups, early and advanced cancers, on the basis of the DNA copy number of the four BAC clones mapped to 1q43, 21q22.2, 16q22.1, and 6p21.1 (Fig 2d). In addition, the examination of the seven BAC clones made it possible to accurately differentiate between these two histologic types, the intestinal-type and the diffuse-type (Fig 2e). A DNA copy number change in a clone located in 5q13.2 was linked with both node and liver metastasis, and in total 24 clones were identified by method 1.

A statistical analysis of the aCGH data was also performed using the protocol referred to as method 2 to identify the clones for classifying the gastric cancers into two groups with opposite characteristics. The comparison of the frequency of the DCNAs clone-by-clone revealed many BAC clones with statistical differences in their frequency between the tumors with and without node metastasis, between the tumors with and without liver metastasis, between the tumors with and without peritoneal dissemination, and between the intestinal- and diffuse-type cancers. Seven, six, five, and eight BAC clones, for a total of 26 clones, were selected to evaluate for node metastasis, liver metastasis, peritoneal dissemination, and histological type, respectively (Table 2).

**Table 2:** 

BAC ID	Chrom. region	Candidate Gene	Frequency of DCNAs(%)	Frequency of DCNAs(%)	P-value*
**Gain**					
Node metastasis			Positive (n = 60)	Negative (n = 23)	
225	2q24.32	TMEM132D	3	26	0.0005
1308	5q13.2	FCHO2, MGC13034	7	43	0.0005
491	16p13.11	PKD1P3	5	35	0.0006
884	19q13.32	N.i.	5	30	0.0006
Liver metastasis			Positive (n = 6)	Negative (n = 77)	
1322	1p13.2	CHIA, C1orf88, OVGP1	43	3	<0.0001
1271	8p22	N.i.	67	13	0.0016
92	11p15	ST5	43	6	0.0006
Peritoneal dissemination			Positive (n = 19)	Negative (n = 64)	
948	15q22.1	MYO1E, LDHAL6B	22	0	0.0002
155	Xp21.2	XK	35	5	0.0009
Histologic type			Diffuse (n = 42)	Intestinal (n = 41)	
563	10p15.3	DIP2C	7	38	0.0011
911	10q24.2	SPFH1, CHUK, CWF19L1	0	23	0.0013
**Loss**					
Node metastasis			Positive (n = 60)	Negative (n = 23)	
936	8p21.2	DOCK5, GNRH1, KCTD9, CDCA2	3	23	0.0023
1158	8p21.1	EXTL3	3	21	0.0023
878	13q31.2	SLITRK5	13	47	0.0007
Liver metastasis			Positive (n = 6)	Negative (n = 77)	
877	1p32.3	LRP8,	50	5	0.0006
1386	7q35	CNTNAP2,	43	4	0.0001
309	21q22.3	HSF2BP, KIAA0179,	29	1	0.0002
Peritoneal dissemination			Positive (n = 19)	Negative (n = 64)	
1407	3p26.3	CNTN6	33	6	0.0009
503	3p24.3	UBE2E1	32	6	0.0009
229	4q31.1	MAML3	44	5	<0.0001
Histologic type			Diffuse (n = 42)	Intestinal (n = 41)	
1292	4q34.1	N.i.	17	53	0.0004
1393	5q33.2	TIMD4	15	48	0.0007
91	8p21.3	INTS10	0	24	0.0006
227	14q32.33	IGHV3-22	16	54	0.0001
1122	15q15.3	GANC, CAPN3	2	27	0.0003
1210	17q13.2	ATP2A3, ZZEF1	5	41	0.0001

N.I.; not identified

* χ^2 ^test between two groups

Ratios (log_2_) that exceed >0.25 are considered DNA copy number gains, and ratios (log_2_) that exceed <-0.25 are considered to be DNA copy number losses.

DNA amplification was detected at chromosomal regions 17q21.1(harboring HER-2), 11q13.3 (CCND1), and 11q13 (FGF4) in four, three, and three tumors, respectively, though the amplification was not correlated with any clinicopathological feature of the gastric cancers.

### Validation of the mini-array

**Figure 3:**
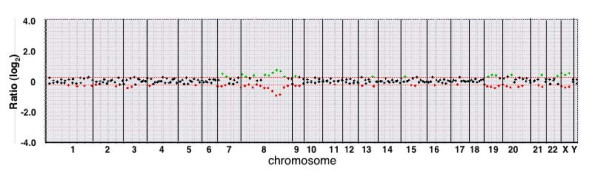
**Array CGH profiles of a case (J91) of gastric cancer in the dye-swap hybridization experiments for the mini-array**. The tumor DNA is labeled with Cy3 or Cy5, and the reference DNA is labeled with Cy5 or Cy3, respectively. Each data point along the ratio plot represents the normalized ratio of the individual clones linearly ordered from chromosome 1 to the Y chromosome. The intensity ratios above or below the threshold are depicted as green or red dots, respectively. Two profiles show a mirror state, though a slight discordance may be present between some dots. The copy number gain is unambiguous for the BAC clones in chromosome 8 in this case.

The dye-swap hybridization experiments revealed that the switching of the dyes did not affect the aCGH profiles (Fig 3). The diagnostic performance of the mini-array was evaluated for the independent set of 30 gastric cancers. Although the density of the dots was less in the mini-array than in the screening 1.4 K arrays, the data by the mini-array analysis were virtually equivalent to those by the screening array (Fig 1c). The copy number of each clone in the mini-array correlated well with that of the corresponding clone in the screening array (average correlation coefficient r = 0.747, ranging from 0.664 to 0.920). First, the mini-array data analysis was made regardless of the clinicopathological features, and then, the aCGH data from the mini-array were checked out against the clinicopathological features of an individual tumor. The diagnostic accuracy was considerably high even in a single analysis method and was not significantly different between the two methods, as shown in Table 3. However, the combination of the two analytical methods slightly improved the accuracy for all parameters except for the histological typing (Table 3). The overall diagnostic accuracy was as follows: 66.7% (sensitivity; 0.95, and specificity; 0.0) for node metastasis, 86.7% (sensitivity; 0.67 and specificity; 0.89) for liver metastasis, 86.7% (sensitivity; 0.20, and specificity; 1.0) for peritoneal dissemination, and 96.7% (sensitivity; 1.0, and specificity 0.67) for the depth of tumor invasion (Table 3). Nine of ten cancers misclassified by the mini-array were false positives, and one of them was a false negative with regard to the evaluation of node metastasis. As for the estimation of liver metastasis, four cancers were misclassified; three of them were false positives and one was a false negative. As for the estimation of peritoneal dissemination, all of the misclassified cancers were false negatives (Table 3). As for the estimation of the depth of tumor invasion, only a single case was misclassified by the mini-array and it was overdiagnosed (Table 3).

**Table 3:** Diagnostic accuracy of the mini-array for an independent series of 30 gastric cancers

	Node Metastasis	Liver Metastasis	Peritoneal Dissemination	Advanced cancers
Accuracy				
Method1	19/30 (63.3%)	27/30 (90.0%)	25/30 (83.3%)	29/30 (96.6%)
Method 2	20/30 (66.7%)	26/30 (86.7%)	26/30 (86.3%)	-
Overall Accuracy	20/30 (66.7%)	26/30 (86.7%)	26/30 (86.7%)	29/30 (96.7%)
Sensitivity	20/21 (0.95)	2/3 (0.67)	1/5 (0.20)	27/27 (1.0)
Specificity	0/9 (0.0)	24/27 (0.89)	25/25 (1.0)	2/3 (0.67)
False positive	9/9 (1.0)	3/27 (0.11)	0/25 (0.0)	1 (over-diagnosis)
False negative	1/21 (0.05)	1/3 (0.33)	4/5 (0.80)	0 (under-diagnosis)

### Effects of intratumoral heterogeneity on the diagnostic performance of the mini-array

A slight variation in the DNA copy number of the BAC clone was observed between the samples taken from a single tumor. Regardless of whether the samples provided a correct diagnosis, however, the diagnosis hardly differed between the four samples from the same tumor (Table 4). In particular, there were no BAC clones with a copy number variation affecting the estimation of liver metastasis and peritoneal dissemination in all tumors. Occasionally there were tumors in which one or two clones affecting the diagnosis were present for one or two items (Table 4). In one early cancer (invasion into the submucosal layer), however, one of four tissue specimens correctly estimated the tumor as an early cancer, while the others estimated it as an advanced cancer. In another case, three of the four tissue specimens correctly estimated a tumor as an advanced cancer, while the other estimated it as an early cancer. However, the overall diagnostic accuracy (in the combination of methods 1 and 2) was virtually unaffected by the intratumoral heterogeneity.

**Table 4:** Intratumoral genomic heterogeneity and diagnostic performance of the mini-array

	Case No.					Total
		
	S39	B17	C22	I87	S50	
**Method 1**						
Node metastasis	-	+ (1)	-	-	+ (2)	3/20
Liver metastasis	-	-	-	-	-	0/20
Peritoneal dissemination	-	-	-	-	-	0/20
Early or advanced	+ (1)	-	-	+ (1)	-	2/20
Histologic type*	+ (1)	-	-	-	+ (2)	3/20
**Method 2**						
Node metastasis	-	-	-	+ (1)	-	1/20
Liver metastasis	-	-	-	-	-	0/20
Peritoneal dissemination	-	-	-	-	-	0/20
Histologic type*	-	-	-	+ (1)	-	1/20

The difference in diagnosis was rare between four samples from the same tumor. In particular, there were no BAC clones affecting the estimation of liver metastasis and peritoneal dissemination in all of the samples.

-; Absence of intratumoral genomic heterogeneity affecting diagnosis

+; Presence of intratumoral genomic heterogeneity affecting diagnosis (the number of clones different from others)

* Intestinal or diffuse type

## Discussion

The evaluation of the disease state before treatment is necessary to appropriately select the optimal treatments with a high therapeutic efficiency and high quality of life for each patient with gastric cancer. With this view, the genomic changes characterizing gastric cancers have been investigated [5-10,13]. The chromosomal regions with frequent copy number aberrations in gastric cancers were easily detected by aCGH [5,7,18,19], and some of them were reported as a genomic marker associated with peritoneal dissemination [20] and node metastasis of gastric cancer [6,18,19,21]. However, the examination of only a few genomic markers is insufficient for the precise estimation of the disease state. Alternatively, the combination of the genomic markers with a strong diagnostic impact may allow for the precise estimation of the disease state in each case. The present study demonstrated that the comparison of the DNA copy number of each BAC clone between the two groups of tumors with opposite characteristics identified the BAC clones linked with the disease states including node metastasis, liver metastasis, peritoneal dissemination, depth of tumor invasion, and histological type of gastric cancer. Two independent data analysis methods selected the BAC clones allowing for the reliable classification of 83 gastric cancers (training samples) into two tumor groups with opposite characteristics. In total, 50 BAC clones were selected as genomic markers for estimating the disease states of the gastric cancers. With the exception of a single clone located in 5q13.2 these clones were associated with only a single characteristic of the disease states. The copy number loss of the clone in 5q13.2 was frequent in the gastric cancers with neither node nor liver metastasis, but the converse was not true. Therefore, although the genetic mechanisms are partly shared by the lymph node and liver metastases of gastric cancer, it is convenient to think that the genomic alterations associated with tumor metastasis are basically different between the target organs, as explained by the well-known 'seed and soil' theory. The BAC clones involved in tumor metastasis are obviously different from the clones involved in the peritoneal dissemination of tumors. These BAC clones identified can be biomarkers to estimate the disease state by definition [22]. At present, however, it is uncertain whether the genes located in these BAC clones are truly involved in the invasion, metastasis or dissemination of gastric cancer, and how the genes participate in the node metastasis, liver metastases or peritoneal dissemination of gastric cancer.

Microarray technologies are being applied to cancer diagnosis and the prognostic prediction in malignancies. A microarray-based gene expression analysis has been proposed to estimate the prognosis of cancer patients [23,24], and a small number of specific microarrays have been developed for this purpose [22,23].

In this study, a gastric cancer specific mini-array was spotted with 138 BAC clones including 50 clones chosen by the screening of 83 cancers. The number of spots in the mini-array was reduced to one-tenth in comparison to the original 1.4 K arrays that were used for the screening of the DNA copy number changes in this study. Furthermore, the number of spotted probes was much less in the present mini-array than in the gene expression type arrays such as the MammaPrint [25]. The mini-array developed in this study allowing the estimation of the disease states based on the DCNAs is unique. This is the first report of the development of a BAC mini-array in order to estimate the disease state of cancer. The diagnostic performance of the mini-array was evaluated for a blinded set of 30 gastric cancers independent of the training set of 83 cases.

The mini-array allows for the automatic differentiation between the tumors with and those without a specific characteristic. The overall accuracy for the blinded-sample set was 66.7% (sensitivity; 0.95, and specificity; 0.0) for node metastasis, 86.7% (sensitivity; 0.67 and specificity; 0.85) for liver metastasis, 86.7% (sensitivity; 0.67, and specificity; 0.89) for peritoneal dissemination, and 96.7% (sensitivity; 0.20, and specificity 1.0) for the depth of tumor invasion. The mini-array provides a guideline for the optimal treatment of each patient with gastric cancer. Early cancers with a node-negative status are an indication for EMR or ESD. Therefore, information concerning the nodal state and the depth of tumor invasion is primarily essential for the determination of the treatment methods and can improve the quality of life for cancer patients. In the differentiation between the early and advanced cancers, the diagnostic accuracy was as high as 96.7% (sensitivity; 1.0, and specificity 0.67). However, the diagnostic accuracy for nodal metastasis was not high; the false positive and false negative rates were 100% and 5%, respectively. These findings indicate the validity of the practical use of the mini-array to determine the optimal treatment methods. However, the diagnostic accuracy, specificity and sensitivity of the mini-array for the blinded samples may not completely meet the requirement of surgeons at present. The mini-array needs to be revised to improve the diagnostic performance. The number of tumors examined in this study was limited, and large-scale studies may identify the BAC clones with a stronger impact on the differentiation between the tumors with and without specific features.

CGH is technically applicable to biopsy specimens as well as surgical ones, because a sufficient amount of genomic DNA can be readily obtained from the biopsy specimens [26]. Before the application of the mini-array to the biopsy specimens, however, the issue of intratumoral heterogeneity must be investigated from the viewpoint of diagnostic accuracy [27]. The effects of the intratumoral heterogeneity on the diagnostic performance of the mini-array were examined using four tissue specimens taken from different parts of a cancer. The diagnosis by the mini-array analysis was hardly different between the four tissue specimens taken from a tumor. This indicates that biopsy specimens as well as surgical ones can be applied to the mini-array for the purpose of estimating the disease states of gastric cancer. Therefore, the mini-array can be added to a list of the laboratory examinations which can be used for the diagnosis of gastric tumors and to also select the optimal therapy.

## Conclusion

We made the mini-array in order to estimate the degree of node metastasis, liver metastasis, peritoneal dissemination, depth of mural invasion, and the histologic type of gastric adenocarcinoma with a high degree of accuracy.

## Abbreviations

BAC: bacterial artificial chromosome; CGH: comparative genomic hybridization; EMR: endoscopic mucosal resection; ESD: endoscopic submucosal dissection.

## Competing interests

The authors declare that they have no competing interests.

## Authors' contributions

TM, SK and TA were responsible for the majority of the molecular genetic studies involving the screening of the gastric adenocarcinomas. TU and AA collected the patient samples and the clinical data in the study. MN and AO did the statistical analyses. KS participated in the study design and helped in drafting the manuscript. SJY contributed the preparation of the mini-array.

The authors read and approved the final manuscript.

## Pre-publication history

The pre-publication history for this paper can be accessed here:



## Supplementary Material

Additional file 1 **Array CGH data.** This file includes the array CGH data for 83 gastric cancers. Click here for file
